# Homology and functions of inner staminodes in *Anaxagorea javanica* (Annonaceae)

**DOI:** 10.1093/aobpla/plaa057

**Published:** 2020-11-10

**Authors:** Bingxin Li, Fengxia Xu

**Affiliations:** 1 Key Laboratory of Plant Resource Conservation and Sustainable Utilization, South China Botanical Garden, Chinese Academy of Sciences, Guangzhou, China; 2 Center of Conservation Biology, Core Botanical Gardens, Chinese Academy of Sciences, Guangzhou, China; 3 College of Life Sciences, University of Chinese Academy of Sciences, Beijing, China; 4 Guangdong Provincial Key Laboratory of Digital Botanical Garden, South China Botanical Garden, Chinese Academy of Sciences, Guangzhou, China

**Keywords:** *Anaxagorea javanica*, Annonaceae, homology, inner staminode, modified stamen, pollination function

## Abstract

Inner staminodes are widespread in Magnoliales and present in *Anaxagorea* and *Xylopia*, but were lost in the other genera of Annonaceae and have no counterparts in derived angiosperms. The coexistence of normal stamens, modified stamens and inner staminodes in *Anaxagorea javanica* is essential to understand the homology and pollination function of the inner staminodes. *Anaxagorea javanica* was subjected to an anatomical study by light and scanning electron microscopy, and the chemistry of secretions was evaluated by an amino acid analyser. Inner staminodes have a secretory apex, but do not have thecae. They bend towards either tepals or carpels at different floral stages, and function as a physical barrier preventing autogamy and promoting outcrossing. At the pistillate phase, the exudates from the inner staminodes have high concentration of amino acid, and provide attraction to pollinating insects; while abundant proline was only detected in stigmas exudates, and supply for pollen germination. Modified stamens have a secretory apex and one or two thecae, which are as long as or shorter than that of the normal stamens. As transitional structures, modified stamens imply a possible degeneration progress from normal stamens to inner staminodes: generating a secretory apex first, shortening of the thecae length next and then followed by the loss of thecae. The presence of modified stamens together with the floral vasculature and ontogeny imply that the inner staminodes are homologous with stamens.

## Teaser

Inner staminodes are widespread in Magnoliales and present in *Anaxagorea* and *Xylopia*, but were lost in the other genera of Annonaceae and have no counterparts in derived angiosperms. This paper demonstrates that the inner staminodes are homologous with stamens in *Anaxagorea* by documenting the existence of modified stamens with reduced thecae and secretory structures, and similar vasculature and phyllotactic continuity of stamens and staminodes as well. This paper also provides original data on floral phenology and chemical exudates of the staminodes and stigmas, which confirm the function of the staminodes during pollination in preventing autogamy.

## Introduction

The Annonaceae is a pantropical family comprising 107 genera and about 2400 species, which is the most species-rich group of the Magnoliales and is one of the largest families of the basal angiosperms (Guo *et al.* 2017). In Annonaceae, two distinct types of staminodes (sterilized stamens) are consequently identifiable based on their position within the flower (Ronse De Craene and Smets 2001; Ronse De Craene 2007): the inner staminodes situated between the functional stamens and carpels, and the outer staminodes, situated between the petals and stamens, are much more common in Annonaceae (Saunders 2010), appearing in *Monanthotaxis* (Ronse De Craene and Smets 1990), *Fusaea*, *Uvaria* (Van Heusden 1992), *Xylopia*, *Orophea* (Keßler 1988) and *Pseuduvaria* (Su *et al.* 2008). Inner staminodes are rare in derived groups, but they are widespread in basal angiosperms (Schodde 1969; Endress 1980, 1984; Endress and Lorence 2004; Staedler *et al.* 2007, 2009; Rohwer 2009; Staedler and Endress 2009), and the presence of inner staminodes may be plesiomorphic within Magnoliales, although they do not occur in Magnoliaceae, Myristicaceae or Annonaceae other than *Anaxagorea* and *Xylopia* (Maas and Westra 1984; Van Heusden 1992; Doyle and Le Thomas 1996; Sauquet *et al.* 2003; Doyle and Endress 2010). *Xylopia* is unique in having both outer and inner staminodes and probably represents an independent evolution of staminodes within the Annonaceae, as the genus is not closely allied to any of the other genera with staminodes (van Heusden 1992; Doyle and Le Thomas 1996; Johnson and Murray 2018).

*Anaxagorea*, the only genus in Anaxagoreoideae, comprises around 30 species with a disjunct distribution in tropical America and Southeast Asia (Maas and Westra 1984, 1985; Maas *et al.* 1986; Van Heusden 1992; Chatrou *et al.* 2012). Studies combining morphological and molecular analysis indicated that *Anaxagorea* is the sister group of the remaining taxa in Annonaceae (Doyle and Le Thomas 1994, 1996; Doyle *et al.* 2000; Sauquet *et al.* 2003). Most species in *Anaxagorea* (except for *A. brevipedicellata* and *A. luzonensis*) possess inner staminodes, which may or may not have a secretory apex (Maas and Westra 1984, 1985; Scharaschkin and Doyle 2005, 2006; Endress and Armstrong 2011).

Since the inner staminodes have no counterparts in more advanced clades of angiosperms (Endress 1984), there have been few reports concentrating on the origin, function and evolution of the inner staminodes. In *Anaxagorea*, the closed pollination chamber during anthesis makes it difficult to directly observe the inner staminodes and movements of pollinators inside (Webber 2002; Braun and Gottsberger 2011; Gottsberger 2016). Saunders (2010) interpreted that inner staminodes with secretory apex were homologues of stigmas, by comparing the scanning electron micrographs of stamens, inner staminodes and carpels, based on research of Scharaschkin and Doyle (2006). However, it seemed insufficient to define the homology of the extremely specialized inner staminodes without structural data. Although some reports postulated that inner staminodes act as a physical barrier and prevent the possibility of autogamy, experiments to clarify their function were very few and indirect (Maas-van de Kamer 1993; Webber 2002; Saunders 2010; Gottsberger 2016). Based only on the absence of gnawing marks on the staminodes and stigmas and without any analysis of the chemical composition of the exudate, Gottsberger (2016) speculated that mucus secreted by the inner staminodes or stigmas might be involved in offering rewards for pollinators (Webber 1996; Braun and Gottsberger 2011; Teichert *et al.* 2011).

In the present studied species *Anaxagorea javanica*, the outer stamens are normal with two thecae and lack a secretory apex. The inner stamens are sterile with a secretory apex and lack thecae, which is consistent with the previous reports (Maas and Westra 1985; Scharaschkin and Doyle 2006). Furthermore, *A. javanica* has one or two modified stamens with a secretory apex and one or two thecae, located between the normal stamens and the inner staminodes. *Anaxagorea javanica* proves to be an excellent species to investigate the homology and functions of the inner staminodes. In the present study, we carefully documented floral phenology and pollinators of *A. javanica*, and analysed the content of exudates from inner staminodes and stigmas, respectively, at the pistillate and staminate phase, with the aim to elucidate the pollination function of the inner staminodes in *A. javanica*. We also tried to clarify the homology of inner staminodes based on the floral anatomy and development, and histological tests comparing the inner staminodes with the modified stamens, normal stamens and carpels. The study provides new insights to understand the evolutionary trends of stamens in Annonaceae, even in Magnoliales.

## Materials and Methods

### Study site

Observations were made in Xishuangbanna Tropical Botanical Garden of the Chinese Academy of Sciences (21°52′N, 101°39′E) in China, Yunnan province. Mature flowers of *A. javanica* were fixed immediately in FAA (70 % alcohol, formaldehyde and glacial acetic acid in a ratio of 18:1:1).

### Phenological observations

Field-based experiments of pollination ecology of *A. javanica* were conducted from September 2018 to October 2019, covering two flowering periods. Thirty flower buds from two individuals of *A. javanica* were tagged and monitored every day until petal opening. Photographic observations were then taken at 2-h intervals afterwards until the end of the staminate phase. The pollinators were observed and photographed using a Zeiss-Smartzoom5 ultra depth of field stereoscopic microscope (Zeiss, Germany).

### Light microscopy

Fixed flower buds, stamens, modified stamens, inner staminodes and pistils were dehydrated in an ethanol series, embedded in paraffin wax and then sectioned at 8 μm. After staining with haematoxylin, Safranin O and Fast Green, iodine-potassium iodide, mercury bromophenol blue and Sudan black, these sections were observed and photographed using a Leica-DM5500B light microscope (Leica, Germany).

### Scanning electron microscopy

Fixed series of flower buds, stamens, modified stamens, inner staminodes, carpels and pollen grains from the normal stamens and the modified stamens were dehydrated using an ethanol series to absolute ethanol. The materials were then critical point-dried, mounted onto scanning electron microscope (SEM) stubs using double-sided adhesive tape and coated with platinum using a JFC-1600 coater. The dried materials were examined using a SEM JSM-6360 cold field emission at 20 kV (JEOL, Tokyo, Japan).

### Pollen germination

Pollen grains were collected from the normal and modified stamens in 10 staminate-phase flowers from two different individuals, and incubated in 100 μL of 10 % sucrose solution on cavity slides within closed Petri dishes for 24 h at ambient temperature (Dafni 1992).

### Stigmatic exudate chemistry

Amino acid extraction methods were adopted from the procedure described by Lau *et al.* (2017). The amino acid composition of the exudates from stigmas and staminodes from the pistillate phase and staminate phase in *A. javanica* were similarly determined using exudate pooled from 10 pistillate-phase and staminate-phase flowers. After being vacuum dried and weighed, 300 μL 0.1M HCl was added to Eppendorf tube containing exudates and ethanol was added to achieve a final concentration of 80 % alcohol. The samples were then vortexed and incubated for 30 min at ambient temperature, and then centrifuged at 3000 rpm for 20 min. The supernatant was vacuum-dried and 200 μL 0.1 M HCl added, then filtered through a 0.22-μm membrane and injected into a Sykam S433 amino acid analyser (fitted with a 150 × 4.6 mm column).

## Results

### Floral phenology

*Anaxagorea javanica* exhibits an annual mode of flowering and has its flowering period from the beginning of September to the end of October. Here, observations are grouped into three phases, viz. pistillate phase, interim phase and staminate phase.

**Figure 1.**
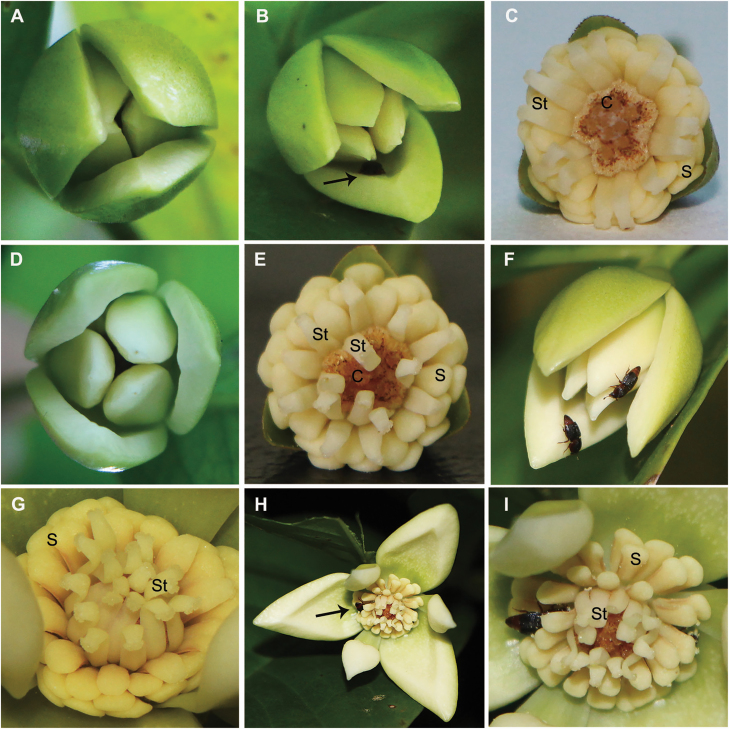
Floral phenology of *A. javanica* during anthesis. (A) The petals of *A. javanica* are slightly opened. (B) Nitidulid beetles visiting gaps between petals at the pistillate phase. (C) The inner staminodes bend towards the stamens and away from the pistils at the pistillate phase. (D) Flower of *A. javanica* at the interim phase. (E) The inner staminodes start bending towards pistils at the interim phase. (F) Flower of *A. javanica* at the beginning of the staminate phase. (G) The inner staminodes are curved over the stigmas at the staminate phase. (H) The petals are widely opened at the end of staminate phase; pollinator (arrow) enters a gap between the petals. (I) There are several *Colopterus* spp. (Nitidulidae) dusted with pollen inside of the flower at the end of the staminate phase. C, carpel; S, stamen; St, staminode.

**Figure 2.**
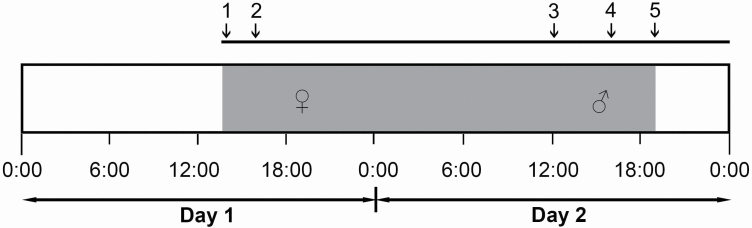
Timing of anthesis of *A. javanica*. (1) Flowers spreading their petals. (2) Initiation of scent production. (3) Initiation of staminate stage. (4) Arrival of pollinators. (5) The end of the staminate stage.

Pistillate phase (ca. 12 h) (Fig. 1A–C): The petals open just slightly by narrow slits at ca. 1400 h (Fig. 1A). Flowers enter the pistillate phase where stigmatic receptivity increases from late afternoon (ca. 1700 h) to the next day (ca. 500 h; Fig. 2). During this stage flowers emit a fruity, banana-like odour, which is particularly strong in the late afternoon to early evening, around 1700 to 2000 h (Fig. 1B). Stigmas turn greenish-yellow and the receptivity is indicated by the secretion of a sticky exudate upon the stigmas. At the same time, the staminodes are covered by a gelatinous exudate and bend towards the stamens and away from the pistils (Fig. 1C).

Interim phase (ca. 11 h) (Fig. 1D and E): The odour of the flowers decreases from ca. 500 h to ca. 1600 h and almost no odour is emitted towards the end of the interim phase (Fig. 2). The stigmatic receptivity of the flowers is reduced during this stage, and the stigmas turn to yellowish brown and become dry (Fig. 1E). The exudate of the inner staminodes slowly dries up as they start to bend towards and enclose the pistils (Fig. 1E).

Staminate phase (ca. 3 h) (Fig. 1F–I): Flowers begin to emit a sweet odour ca. 1600 h. The inner staminodes eventually enclose the pistils and then the thecae dehisce, providing large amounts of pollen for pollinators (Fig. 1G and H). Around 1900 h at the end of the staminate phase, the pollination chamber momentarily opens, and petals abscise from the receptacles in less than a minute, and then petals, stamens and inner staminodes are gradually lost (Figs 1H, I and 2).

**Figure 3.**
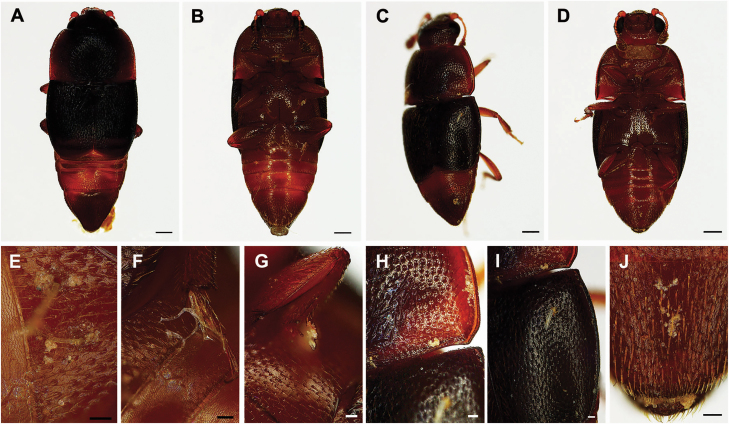
Microphotographs of the two putatively most important pollinators. (A, B, E–G) *Colopterus* spp. (Nitidulidae). (A) Dorsal view. (B) Ventral view. (E–G) Detail of pollen sticking to the abdomen and legs of *Colopterus* spp. (C, D, H–J) *Epuraea* spp. (Nitidulidae). (C) Dorsal view. (D) Ventral view. (H–J) Detail of pollen sticking to the abdomen and legs of *Epuraea* spp. Scale bars: A–D = 250 μm; E–J = 50 μm.

One or two nitidulid beetles of the genera *Colopterus* or *Epuraea* were observed arriving on the petals at the staminate phase (around 1630 h), entering the pollination chamber, and then departing the flowers at the end of the staminate phase (around 1900 h; Figs 1F, I and 3A–D). They were later found on the petals of another flower at the pistillate phase (around 1800 h; Fig. 1B). Large numbers of pollen grains stick to the abdomens and legs of the *Colopterus* spp., which were covered with a great deal of bristly hairs (Fig. 3E–G). Abundant pollen was observed in *Epuraea* spp. on the dorsal shell and ventral tail covered with bristles (Fig. 3H–J).

### Stamen type and morphology

**Figure 4.**
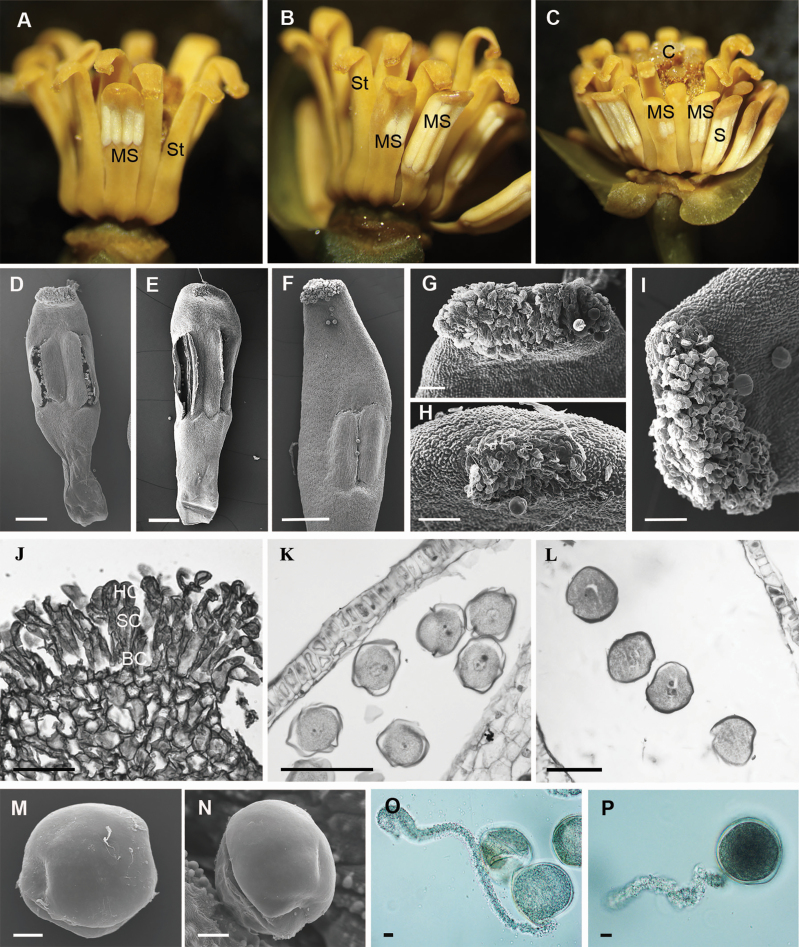
The morphology of modified stamens and pollen grains from the normal stamens and the modified ones. (A–C) Modified stamens located between the normal stamens and the inner staminodes. (A) Flowers with one modified stamen possessing a secretory apex and two shorter thecae than in the normal stamens. (B, C) Flowers with two adjacent modified stamens covered by secretory structures at the apex: one with two thecae of the usual length or shorter thecae, the other with one shorter theca. (D, E) Modified stamens with two shorter thecae than in the normal stamens. (F) Modified stamen with one shorter theca. (G–I) The apex of modified stamens covered by secretory structures. (J) Longitudinal microtome sections of the apex of inner staminodes, showing one basal cell, three stalk cells and one head cell. (K, M) Pollen grains from a normal stamen. (L, N) Pollen grains from a modified stamen. (O) Germinating pollen from a normal stamen producing a pollen tube. (P) Germinating pollen from a modified stamen producing a pollen tube. BC, basal cell; C, carpel; HC, head cell; S, stamen; SC, stalk cell; MS, modified stamen; St, staminode. Scale bars: D–F = 500 μm; G–I = 100 μm; J–L = 50 μm; M–P = 10 μm.

Three types of stamens were observed in *A. javanica*, viz. normal stamens, modified stamens and inner staminodes. In an individual flower, around 33–36 normal stamens are located in the outer portion of androecium (Fig. 7A). They are laminar and have two thecae, extrorsely dehiscing and completely embedded within a tongue-shaped connective (Fig. 7P and Q). About 15–20 staminodes are located in the inner portion of the androecium (Figs 1C, E, H, 7B and C). The long and narrow inner staminodes are S-shaped and lack thecae (Fig. 7R and S). The apex of the inner staminodes is densely covered by secretory structures (Figs 7O and 8F). In each flower of *A. javanica* examined, only one or two modified stamens were found between the normal stamens and the inner staminodes, with the apex covered by secretory structures and the thecae dehiscing extrorsely (Fig. 4A–J). In the individual flowers with only one modified stamen, the two thecae are as long as or one-fourth to three-fourths shorter than those in the normal stamens (Fig. 4A, D and E). In the flowers with two modified stamens, they are always adjacent to each other, one is similar in organization to a single modified stamen (Fig. 4B–E), the other has only one much smaller theca (usually one-fifth the length of the thecae in the normal stamens) (Fig. 4B, C and F) and was not found on its own in any individual flower. Pollen grains from both the normal stamens and the modified stamens are ellipsoidal monads and the exine ornamentation is psilate (Fig. 4K–P). The pollen germination rate is 46 % in normal stamens, while the pollen germination rate can attain 51 % in modified stamens.

### Floral vasculature

**Figure 5.**
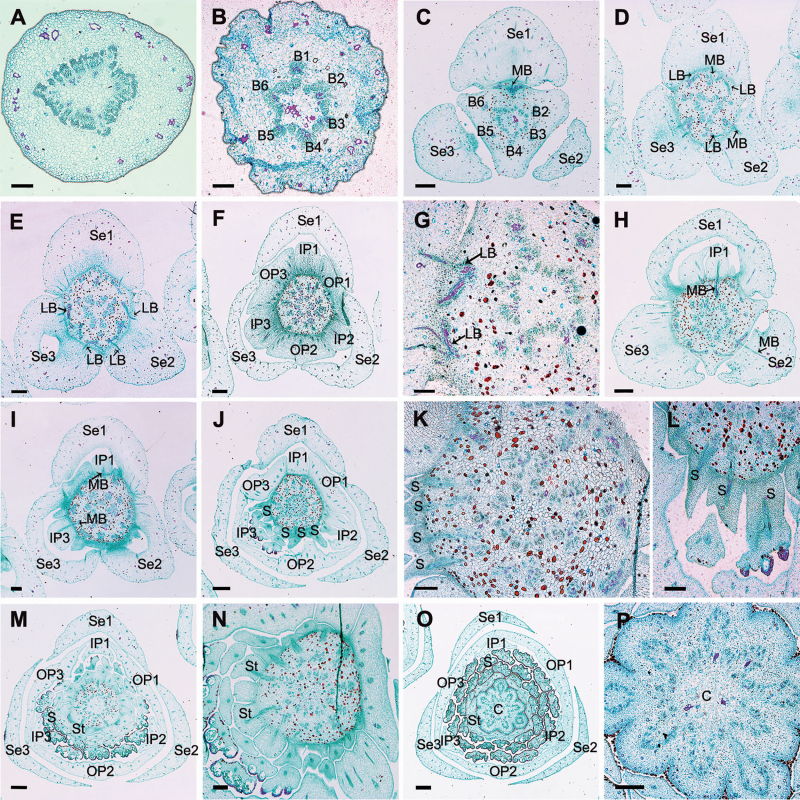
Transverse sections showing the floral vasculature of *A. javanica.* (A–P) Sections shown in sequence from base to apex. (A) Section through pedicel, showing stele. (B) Base of receptacle, showing six groups of vascular bundles. (C–E) Level where the sepals are connected to the receptacle, showing their median bundles and lateral bundles. (F, G) Level where outer petals are connected to the receptacle, showing their median bundles and lateral bundles. (H, I) Level where sepals and inner petals are connected to the receptacle, showing their median bundles and lateral bundles. (J–L) Top of the receptacle, showing vascular bundles leading to stamens. (M, N) Top of the receptacle, showing vascular bundles leading to inner staminodes. (O) Section through flower, above receptacle, showing positions of carpels, inner staminodes, stamens, petals and sepals. (P) Top of receptacle, showing vascular bundles leading to carpels. B, bundle; LB, lateral bundle; MB, median bundle; C, carpel; S, stamen; St, staminode; Se, sepal; OP, outer petal; IP, inner petal. Scale bars: A, B, G, K, L, N, P = 200 μm; C–F, H–J, M, O = 500 μm.

Serial sections through the receptacle of *A. javanica* reveal the pattern of vasculature supplying each perianth organ. The stele in the pedicel (Fig. 5A) diverges into six groups of vascular bundles at the base of the receptacle (labelled B1–6 in Fig. 5B): three of these bundle clusters (B1, B3 and B5 in Fig. 5B) fuse with the median bundle of the sepals (MB in Fig. 5C–E) and the vasculature supplying the inner petals (Fig. 5H and I); the other three groups (B2, B4 and B6 in Fig. 5B) connect with the vasculature of the outer petals (Fig. 5F and G) and two lateral bundles feeding adjacent sepals (LB in Fig. 5D and E). Each stamen and staminode receives only one vascular trace (Fig. 5J–N). Each of the carpels has three distinct longitudinal vascular bundles, a dorsal bundle and two ventral ones, and there are horizontal connections between them (Fig. 5O and P).

**Figure 6.**
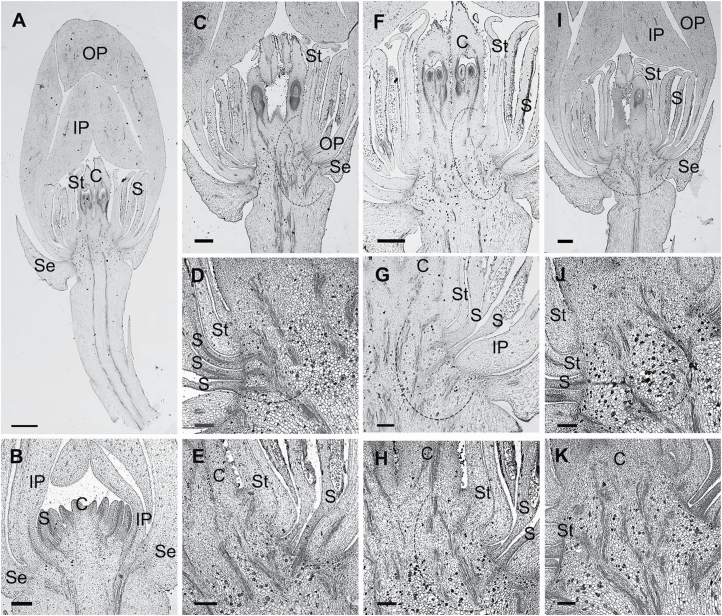
Longitudinal sections of a flower of *A. javanica*. (A) Longitudinal section of the *A. javanica*. (B) Petal and septal bundles located in the outer whorl. (C–E) Stamens and inner staminodes bundles have a common origin. (F–H) Inner staminode bundles fuse with lateral carpellary bundles. (I–K) Carpels are vascularized by synlateral and dorsal bundles. C, carpel; S, stamen; St, staminode; Se, sepal; OP, outer petal; IP, inner petal. Scale bars: A, C, F, I = 500 μm; D, E, G, H, J, K = 100 μm.

The longitudinal sections of *A. javanica* show the positions of carpels, inner staminodes, stamens, petals and sepals (Fig. 6A). The perianth cortical vascular system is located in the outer layer (Fig. 6B). The vasculature of the inner staminodes splits from the basal traces of the free stamen bundles (Fig. 6C–E), and then the inner staminode bundles fuse with middle bundles supplying carpels (Fig. 6F–H). With the ventral bundles ending in the apex of the ovary, the dorsal carpellary bundles follow the narrowing of the ovary and enter the style where they continue acropetally through the style as three distinct bundles before ending blindly in the base of the stigma (Fig. 6I–K). The median bundle supplies the ovary, which has two lateral ovules with the placentae at the base (Fig. 6F and I).

### Staminal and pistillary ontogeny

**Figure 7.**
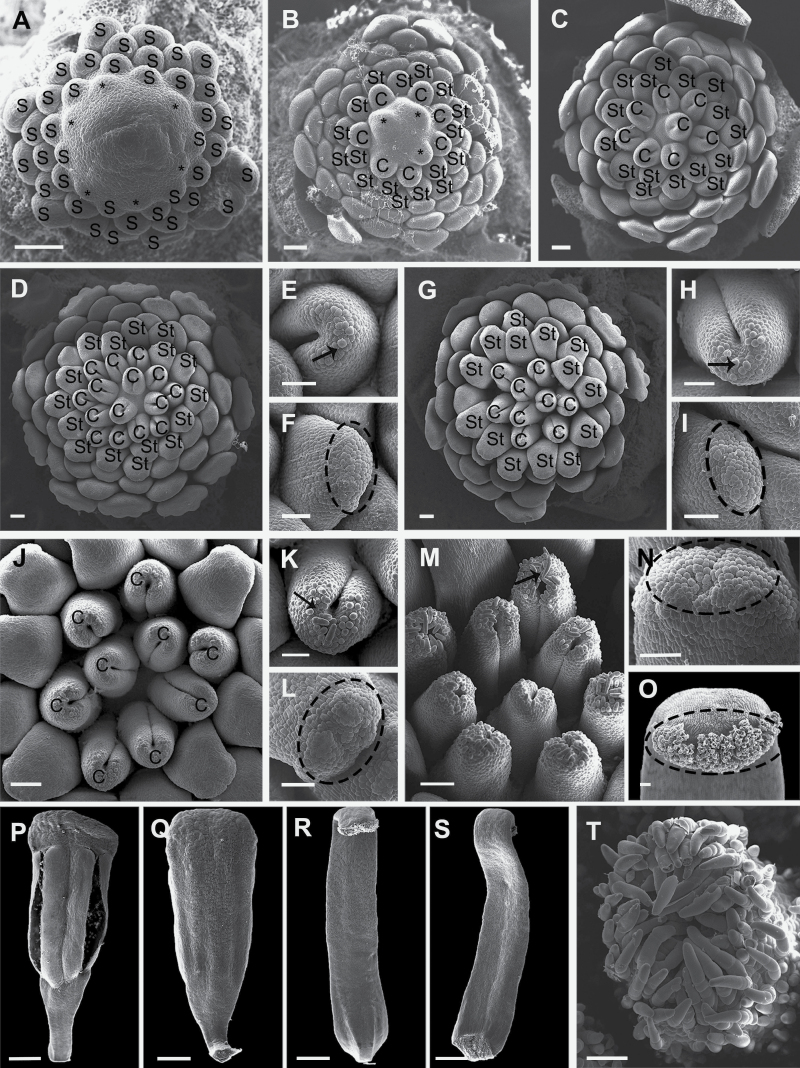
Staminal and pistillary ontogeny of *A. javanica* observed with a scanning electron microscopy. (A) Initiation of the first whorl of inner staminode primordia (asterisks). (B) Initiation of the second whorl of carpel primordia (asterisks). (C) Horseshoe-shaped carpel primordia with developing concavities on the ventral surface. (D–N) Comparative development of the gynoecium and the staminodes. (D) After the inception of the last carpels, the ventral depression deepens and extends to the tip of each carpel. (E) Carpels covered by trichomes (arrow). (F) The top cells of staminodes become bigger than others. (G) Mature flower bud. (H) Multicellular trichomes produced by division (arrow). (I) Cells at the top of inner staminodes start bulging out. (J) Nine carpels in the flower. (K) Glandular hairs on top of the carpels increase in size (arrow). (L) Cells at the top of the inner staminodes start bulging out. (M) Glandular hairs begin to seal the carpels (arrow). (N) Glandular hairs of the inner staminodes become distinct. (O) Inner staminodes have a secretory apex. (P) A stamen from the dorsal side. (Q) A stamen from the ventral side. (R) A staminode from the dorsal side. (S) A staminode from the ventral side. (T) The cylindrical stigma densely covered with trichomes. C, carpel; S, stamen; St, staminode. Scale bars: A–O, T = 50 μm; P–S = 500 μm.

Following the forming of normal stamen primordia, the six inner staminode primordia are initiated (Fig. 7A). Further initiation of the inner staminode (9–14) runs in an irregular sequence on the floral apex in a relatively rapid succession. After the initiation of inner staminodes, 9–15 carpels primordia develop rapidly in an irregular sequence at the centre of the floral apex (Fig. 7B–D), and the anthers of normal stamens begin to differentiate at the same time (Fig. 7B). When all flower organs have emerged, the carpel primordia become horseshoe-shaped with developing concavities on their ventral surface (Fig. 7C). The epidermal cells of the inner staminodes are unequal in size and somewhat protrude at the apex, making the surface uneven, while in the normal stamens, the surface of the connective appendage apex is smooth without trichomes (Fig. 7D and F). At this stage, both the capitate and peltate trichomes originate from a single protodermal cell, larger than the neighbouring ones of the stigmas (Fig. 7E). With the development of the flower, the differences between the secretory structures at the apex of the inner staminodes and carpels become more and more obvious (Fig. 7G–N). The cylindrical stigmas become densely covered with filamentous and capitate peltate trichomes (Fig. 7T).

### Trichome development in inner staminodes and stigmas

**Figure 8.**
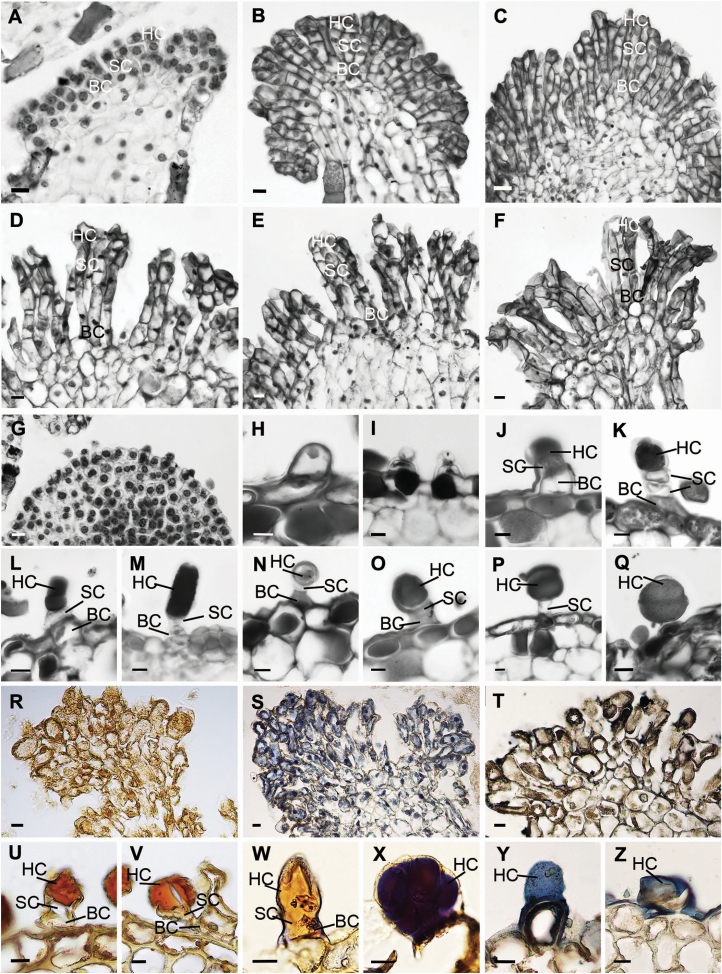
The development of trichomes on the inner staminodes and carpels. (A–F) Longitudinal microtome sections of the apex of inner staminodes, showing the development of secretory structures with the one basal cell, three stalk cells and one head cell. (G–Q) Lateral view of capitate and peltate trichome. (G) The outgrowth of initial cells of stigmas. (H) A vacuolized initial cell. (I) Two sister cells formed after the first periclinal division of the initial cell. (J) Three-cell stage, a capitate trichome showing the vacuolized basal cell. (K) Four-cell stage, a capitate trichome showing the vacuolized stalk cells. (L–M) A capitate trichome with one basal cell, one or two stalk cells and one head cell. (N) A peltate trichome with one basal cell, one stalk cell and multicellular head. (O) A peltate trichome showing the secreted multicellular head. (P, Q) The multicellular head secretions increase. (R) The secretory apex in the inner staminodes are negative to iodine-potassium iodide. (S) The secretory apex in the inner staminodes stained by mercury bromophenol blue for abundance of proteins. (T) The secretory apex in the inner staminodes stained by Sudan black shows small amounts of total lipids. (U) Capitate trichomes are negative to iodine-potassium iodide. (V) Peltate trichomes are negative to iodine-potassium iodide. (W) Capitate trichomes are negative to mercury bromophenol blue. (X) Peltate trichomes head stained by mercury bromophenol blue for abundance of proteins. (Y, Z) Capitate trichomes and peltate trichomes subcuticular space stained by Sudan black for total lipids. BC, basal cell; HC, head cell; SC, stalk cell. Scale bars: A–Z = 10 μm.

At an early stage, the glandular cells of the inner staminodes consist of one basal cell, two stalk cells and one head cell, and present large nuclei and dense cytoplasm (Fig. 8A). After one periclinal division forms three stalk cells, the size of glandular cells in the inner staminodes continues to increase at a successive developmental stage (Fig. 8B–E). At maturity, the trichome cells of the inner staminodes are rectangular with a large nucleus and thin cytoplasm (Fig. 8F). The initial cells of capitate and peltate trichomes of stigmas are spherical and all derive from the epidermis (Fig. 8G). The glandular cells of stigmas continue protruding outward and gradually become oblong (Fig. 8H). Two sister cells are formed after the first periclinal division of the glandular cells (Fig. 8I). The top cell undergoes a periclinal division, producing two daughter cells: the stalk cell and the head cell (Fig. 8J). The secretory cells in the head continue to develop vacuoles, and the secretory cells begin to be filled with secretions (Fig. 8K–M). These trichomes of stigmas contain two types: capitate trichomes with one or two basal cells, one to three stalk cells and one head cell (Fig. 8M), together with peltate ones with one basal cell, one stalk cell and a multicellular head (Fig. 8N and O). The secreting multicellular head continues to increase in peltate trichomes (Fig. 8P and Q). The secretory structures at the top of inner staminodes are negative to iodine-potassium iodide but are positive to mercury bromophenol blue and Sudan black, showing them to be rich in proteins and lipids (Fig. 8R–T). Both capitate and peltate trichomes are negative to iodine-potassium iodide (Fig. 8U and V). Capitate trichomes are negative to mercury bromophenol blue (Fig. 8W), while peltate trichome heads are positive to mercury bromophenol blue for rich proteins (Fig. 8X). The subcuticular space of both capitate and peltate trichomes could be stained by Sudan black for total lipids (Fig. 8Y and Z). Because only one or two modified stamens were present per flower, the development of secretory structures could not be detected; only the mature structure was observed, which consists of one basal cell, three stalk cells and one head cell (Fig. 4J).

### Exudate chemistry at the apex of staminodes and pistils

Total amino acid concentrations at the apex of the inner staminodes and stigmas are 7576.5 and 3594.5 μg g^−1^ at the pistillate stage, and 3512.5 and 2161.1 μg g^−1^ at staminate phase, respectively (Table 1). Nine essential and 22 other amino acids (along with urea) were recorded from the apex of inner staminodes at the pistillate phase: the most abundant of these was asparagine (1549 μg g^−1^; 20.4 % of total amino acid content), followed by alanine (902.5 μg g^−1^; 11.9 %). Nine essential and 23 other amino acids were recorded from the stigmas at the pistillate phase: the most abundant of these is alanine (542 μg g^−1^; 15.1 % of total amino acid content), followed by hydroxyproline (518.5 μg g^−1^; 14.4 %). Analysis of the apex of inner staminodes and the stigmas at the staminate phase revealed a similar range of amino acids, both with 9 essential and 22 other amino acids recorded. Alanine was found to be the most abundant (946.5 μg g^−1^; 26.9 %) in the inner staminodes, while urea was found to be the most abundant (316.8 μg g^−1^; 14.7 %) in the stigmas. Serine is the second-most abundant amino acid in both inner staminodes (360 μg g^−1^; 10.3 %) and stigmas (246.2 μg g^−1^; 11.4 %). The modified stamens are covered by a small quantity of exudates at the pistillate phase (Fig. 4A–I). Additionally, because there are only one or two modified stamens per flower, the exudate is too scanty to be collected for chemistry tests.

**Table 1.** Amino acid (AA) composition of the exudates from the stigmas and staminodes.

AA	Pistillate phase (μg g^−1^)		Staminate phase (μg g^−1^)	
	Inner staminodes	Stigmas	Inner staminodes	Stigmas
Essential AAs				
1. Threonine	440	34.5	91	186.3
2. Leucine	182.5	119.5	138.5	102.8
3. Histidine	111	23	48	10.3
4. Isoleucine	811	9	105	77.8
5. Valine	148.5	35	106.5	33.3
6. Phenylalanine	99.5	51	70	26.2
7. Methionine	142	341	20	2.2
8. Arginine	46.5	104	119	10.7
9. Lysine	79.5	48	81.5	37.7
Non-essential AAs				
10. Alanine	902.5	542	946.5	48
11. Serine	711	271	360	246.2
12. Tyrosine	493	109	306	203.3
13. Urea	173	61.5	40	316.8
14. Glutamic acid	330	131	55	90.83
15. γ-Aminobutyric acid	403.5	220	175	148
16. Asparagine	1549	85.5	244.5	87.3
17. Phosphoserine	67.5	76.5	165.5	135
18. Aspartic acid	205	60.5	150	77.3
19. Glycine	41.5	11.5	27	8.7
20. α-Aminobutyric acid	45.5	1	4.5	1.7
21. Cystine	93	4	5	10.8
22. β-Aminoisobutyric acid	11.5	13.5	6.5	35.5
23. β-Alanine	5.5	23.5	4.5	27.3
24. Ornithine	10.5	76	126.5	80.5
25. Taurine	58	21	19	7.5
26. Phosphorus ethanolamine	80	121	69	20.8
27. α-Amino-adipic acid	77	161.5	7	4.3
28. Citrulline	10	52.5	5	0.2
29. 3-Methyhistidine	164.5	28.5	1	26.7
30. 1-Methyhistidine	25.5	104	2.5	–
31. Carnosine	59	–	13	56.8
32. Hydroxyproline	–	518.5	–	–
33. Proline	–	136	–	40
Total AAs	7576.5	3594.5	3512.5	2161.1

## Discussion

### Staminodes as a physical barrier preventing autogamy

An 11-h non-reproductive interim phase separating the pistillate and staminate phases in *A. javanica* indicates that there is no temporal overlap between pollen presentation and stigmatic receptivity. At the pistillate stage, the inner staminodes bend towards the tepals and away from the pistils at a right angle, such that beetles penetrating the pollination chamber are canalized towards the region of the carpels, where they can deposit pollen stuck to their bodies. When the flowers come into the staminate stage, anthers shed their pollen, and the inner staminodes incline towards the carpels, sometimes grow a bit longer and even partly cover the stigmas. This makes the way free for the beetles to crawl down to the dehisced stamens and receive a new pollen load before leaving the flower. Owing to the movements of the inner staminodes, the beetles are always being concentrated in the right compartment of the pollination chamber. This has also been reported in other species of *Anaxagorea* (*A. brevipes*, *A. dolichocarpa*, *A. phaeocarpa*), as well as in Eupomatiaceae, Himantandraceae and Degeneriaceae (Endress 1984; Maas-van de Kamer 1993; Webber 1996, 2002; Braun and Gottsberger 2011; Gottsberger 2016). In *A. javanica,* inner staminodes play a key role in flower–animal interactions and act as a physical barrier to prevent the stigmas from receiving their own pollen. Adding the non-sexual interim phase between stigmatic receptivity and pollen presentation as well, outcrossing in *A. javanica* is undoubtedly promoted and the possibility of autogamy is limited.

### Exudate from staminodes and stigmas provides different needs

In both the inner staminodes and stigmas, the content of the exudate is rich in proteins and lipids, but lacks starch. At the pistillate stage, more proteins are in the inner staminodes than in stigmas, and consequently, the total amino acid content in the inner staminodes (7576.5 μg g^−1^) is more than twice that in the stigmas (3594.5 μg g^−1^; Fig. 10). It has been reported that 10 amino acids are essential for insects and cannot be synthesized by the insects themselves, and hence, these amino acids must be obtained through their diet (Haydak 1970; Baker and Baker 1975; Baker 1977; Gottsberger *et al.* 1984). The 10 essential amino acids have previously been recorded in floral nectar. However, in this study, nine of these were detected both in the inner staminode and stigma exudates (only tryptophan is absent). At the pistillate phase, the concentration of the nine essential amino acids in the inner staminode exudate (2060.5 μg g^−1^) is nearly three times higher than that in the stigma (765 μg g^−1^; Fig. 10), with inner staminodes bending towards stamens and pollinators penetrating the pollination chamber. This implies that the exudate from inner staminodes could supply the energetic demands of nitrogen for flower pollinators (Nicolson and Thornburg 2007), because of its high amino acid content, without relying on the absence of gnawing marks on inner staminodes as Gottsberger speculated (2016), or the exudate being sticky (Endress 1984; Endress and Hufford 1989; Armstrong and Irvine 1990). Therefore, the exudate from stigmas may be avoided for consumption by flower visitors. At the staminate phase, the inner staminodes eventually enclose the pistils. The total amino acid content in the inner staminode exudate decreases halfway compared to the pistillate phase (7576.5 μg g^−1^; Fig. 10), and may not be nutritious enough for the insects. The pollinators stayed around the dehisced stamens and nourished themselves on the large amounts of pollen, shed from the normal stamens. Obviously, during anthesis of *A. javanica*, a close interaction operates between the movements, alteration of amino acid content in the exudate and the pollination function for the inner staminodes.

**Figure 9.**
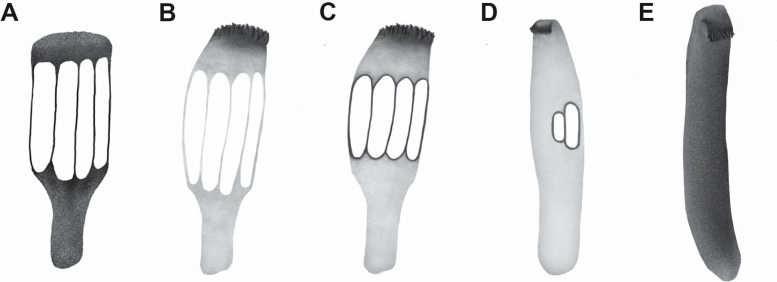
The reductive processes in the androecium, showing the secretory apex generated and thecae reduced. (A) The normal stamen with two thecae, but without secretory apex. (B) The modified stamen with secretory apex and two thecae of usual length. (C) The modified stamen with secretory apex and two thecae one-fourth to three-fourths shorter than that in the normal stamens. (D) The modified stamen with secretory apex and one much smaller theca (usually one-fifth of the normal stamens). (E) The inner staminode with secretory apex, but without thecae.

**Figure 10.**
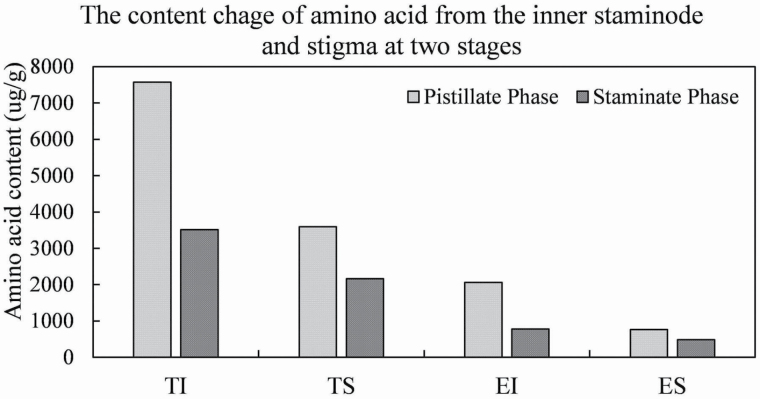
The content change of amino acid from the inner staminodes and stigmas at the pistillate and staminate phase. TI, total amino acid in the inner staminode; TS, total amino acid in the stigma; EI, essential amino acid at the inner staminode; ES, essential amino acid in the stigma.

Despite the fact that both the total amino acid concentration and the nine essential amino acids were much lower in the stigma exudate than in the inner staminodes, proline was only detected in stigmas. Proline has been reported to play a metabolic role in pollen tube growth and supply for pollen germination (Linskens and Schrauwen 1969; Schrauwen and Linskens 1974; Hong-Qi *et al.* 1982), which could be acquired from stigma exudates or pollen (Hong-Qi *et al.* 1982; Lau *et al.* 2017). In *A. javanica*, abundant proline was present in stigma exudate at the pistillate phase. At the end of the staminate phase, the proline content decreased obviously with the reduction of exudate, and the stigma became yellowish brown, indicating that the stigmatic receptivity decreased and the stigma was not suitable for pollen germination at this period. Interestingly, proline was not found in the inner staminode exudate, neither at the pistillate nor the staminate phase. It can be hypothesized that the staminode exudate provides for the needs of pollinating insects, while that of stigma provides for the needs for pollen germination.

### The homology of inner staminodes

*Anaxagorea javanica* possesses a vascular anatomy that is typical of the Annonaceae, comprising an outer perianth cortical vascular system (CVS) with three whorls of vascular traces and the inner corolla whorls that contain the vascular bundle of the stamens, inner staminodes and carpels (Deroin 1989, 1999; Xue and Saunders 2013; Guo *et al.* 2018). The longitudinal sections show that the vascular bundles to the inner staminodes are derived from the basal traces of the free stamen bundles and fused with carpellary bundles from the base. When all the flower organ primordia are developed, the inner staminode primordia are laminar, while the carpel primordia are horseshoe-shaped with developing concavities on the ventral surface. In the inner staminodes, the epidermal cells at the apex protrude somewhat, forming a secreting surface at this region. In stigmas, both the capitate and peltate trichomes originate from a single protodermal cell, larger than the neighbouring ones. In mature flowers, the inner staminodes are S-shaped and the apex is densely covered by secretory structures, providing needs of pollinating insects, being rich in proteins and lipids. The stigmas are densely covered by capitate trichomes, together with peltate ones. The former contain lipids only, and the latter are rich in proteins and lipids, providing needs for pollen germination. Previous studies (Saunders 2010) suggested that the secretory apex of the inner staminodes resembles a modified stigma in *Anaxagorea*. There seems to be no support for this study, because there are obvious differences between the inner staminodes and stigmas in flower vasculature, staminal and pistillary ontogeny, and trichome development.

In the investigated individuals of *A. javanica* (about 50 in total), three types of stamens were observed in one flower. The normal stamens are laminar with smooth and tongue-shaped connective appendages and have two thecae, extrorsely dehiscing. Between the normal stamens and the inner staminodes, one or two modified stamens have a secretory apex and two thecae, as long as or shorter than that in the normal stamens, or sometimes only a single theca (Fig. 9B–D). Inner staminodes lack thecae, and the apex is densely covered by secretory structures. Both the normal stamens and the modified one(s) produce ellipsoidal pollen grains and the exine ornamentation is psilate. In addition, pollen germination rates are nearly equal (51 % for the normal stamens, and 46 % for the modified ones). On the other hand, both the modified stamens and the inner staminodes have a similar secretory apex with one basal cell, three stalk cells and one head cell, and produce sticky exudate at the pistillate phase, although the exudate chemistry of the modified stamens was not available. Obviously, the modified stamens have some characters in common with normal stamens and inner staminodes, and hence could be recognized as the transitional station between the normal stamens and the inner staminodes. From normal stamens to inner staminodes, the degeneration is clear and progressive, generating a secretory apex first, shortening thecae length next and finally losing thecae, one after the other (Fig. 9).

Some morphological differences between the normal stamens and the inner staminodes are present during the developmental process. In normal stamens with two extrorse thecae, the surface at the level of the connective appendage apex is smooth without trichomes, while in the inner staminodes, the thecae abort, but trichomes appear and gradually protrude outward at the apex, forming the secretory structures. However, the inner staminodes are initiated as a regular whorl similar to normal stamen primordia, and the vasculature of the inner staminodes splits from the basal traces of the free stamen bundles. Additionally, because the modified stamens are transitional structures between the normal stamens and the inner staminodes, the inner staminodes should be recognized as homologous with stamens in *A. javanica*, even though they are morphologically and functionally different from normal stamens. As the end result of stamen reduction, the secretory apex and absence of thecae in the inner staminodes were recognized as extreme specializations, also observed in other families of Magnoliales, namely Eupomatiaceae, Himantandraceae, Degeneriaceae (Endress 1984). Maas and Westra (1984) noted the reduction of thecae in the modified stamens but did not report the secretory apex. In the present study, *A. javanica* is the only example in Annonaceae with modified stamens possessing secretory apex, which not only strengthens the staminal origins of the inner staminodes in *A. javanica*, but also provides a clue to recognizing evolutionary trends of stamens in Magnoliales.

## Conclusions

This is the first observation of the occurrence of three types of stamens in *A. javanica*, viz. normal stamens, modified stamens and inner staminodes. Inner staminodes play a role as a physical barrier preventing autogamy and promoting outcrossing, and their exudate provides for the needs of pollinating insects. The presence of modified stamens implicates that the possible transition from normal stamens to inner staminodes may involve a sequence of origin of a secretory apex first, shortening of the thecae next and finally loss of the thecae, one after the other. It also provides a clue to recognize evolutionary trends of stamens in Magnoliales. The transitional modified stamens together with the floral vasculature and ontogeny support the homology of the inner staminodes with normal stamens.

## Notes

**Form & Function. Chief Editor:** Kate McCulloh
